# Multi-stage framework for optimal incorporating of inverter based distributed generator into distribution networks

**DOI:** 10.1038/s41598-024-62708-6

**Published:** 2024-06-10

**Authors:** Shrouk A. Hamza, Mariem Y. Yousef, A. A. Ali, Said M. El Masry, Magdi A. Mosa

**Affiliations:** Electrical Power and Machines Department, Faculty of Engineering, Helwan University, Cairo, Egypt

**Keywords:** Inverter based distributed generator, Hydrogen based distributed generators, Fuel cell, Harmonic power flow, Harmonic pollution, Voltage profile, Electrical and electronic engineering, Power distribution

## Abstract

Recently, hydrogen-based distributed generators (DG) have gained significant attention for modern energy generation systems. These modem DGs are typically outfitted with power electronics converters, resulting in harmonic pollution. Furthermore, increasing the growth of modern nonlinear loads may result in exceeding the harmonic beyond the permitted level. This research proposes a framework for optimal incorporation of inverter-based distributed generation (a fuel cell connected to an AC distribution system) for minimizing power losses, enhancing the voltage profile, and limiting both total and individual harmonic distortion according to the IEEE-519 standard. In addition, for accounting system sustainability, the proposed framework considers load variation and the expected rise in demand. Therefore, the suggested framework comprises three stages, which include fundamental and harmonic power flow analysis. The first stage identifies the optimal size and location of the DG in relation to the base load operating condition. While, with the optimal DG of the first stage, the amount of harmonic pollution may violate the limits during a high level of nonlinear load penetration, as a result, the second stage resizes the DG, considering the connection bus of the first stage, to mitigate the harmonics and optimize the system at a higher level of nonlinear load penetration. Both the first and second stages are performed off-line, while the third stage optimizes the system operation during run time according to loading conditions, harmonic pollution, and the available DG capacity of the previous stages. DG’s harmonic spectrum is represented according to recently issued IEEE 1547-2018 for permissible DG’s current distortion limits. The suggested approach is applied and evaluated using an IEEE 33-bus distribution system for various combinations of linear and nonlinear loads. For run-time operation throughout the day, the presented framework reduces the energy losses from 5.281 to 2.452 MWh/day (about 53.57% energy savings). This saving is associated with voltage profile enhancement without violating the permissible standard levels of harmonics and other system constraints.

## Introduction

Distributed generators (DGs) provide numerous economic, environmental, and technical benefits. Technical benefits include improving the voltage profile due to changing the magnitude and direction of real and reactive power flows. Environmental issues such as pollution reduction through utilization of renewable resources or clean fuel. Hydrogen-based distributed generators, such as fuel cells, have recently attracted substantial interest for modern energy-generating systems^1^. Fuel cells provide various advantages, such as high energy and power density^2^, a clean generator^3^, no mechanical movement, and the ability to provide an ancillary service such as injecting active and reactive power into the grid^4,5^. However, a fuel cell is a DC source, which requires an interface inverter for incorporation into the AC distribution network^2^. These power electronic devices inject a significant amount of harmonic, which causes several power-quality challenges that shall be addressed. Moreover, improper DGs planning (sizing and allocation) might result in increased losses, higher harmonics, or even overvoltage^6,7^.To tackle these challenges, the DG size and location shall be carefully chosen^8^.

DG size and allocation in distribution networks have been investigated in several studies to optimize one or more system variables, such as maximizing the hosting capacity and minimizing the total energy losses^3^, minimizing the real power losses and enhancing the VSI^9^, diminishing both the power losses and the voltage deviation^10^, maximizing DG hosting capacity^11^, minimizing active power losses, reactive power losses, voltage deviation, and maximizing the VSI^12^, minimizing costs and enhancing the economic efficiency of the power systems^13,14^, and minimize fuel cost, real power losses, emission cost, and voltage deviations^15,16^. Also, several types of DGs (wind, PV, and fuel cell) have been optimized for loss reduction and voltage profile enhancement in^9^, energy losses and emission reduction in^17^, and minimizing the injected power into the grid in^18^. In^19,20^, system reconfiguration has been employed with DG allocation to optimize the mix of power losses and voltage deviation. The artificial hummingbird algorithm (AHA) has been introduced in ref^21^ to obtain the optimal sizing and siting of renewable DG in the IEEE-33 bus radial distribution system for reducing total voltage deviations, emissions, costs, and increasing system stability. These studies^3,10,12,17–20^ have not investigated the DG’s reactive power capability, which has a significant impact on voltage profile enhancement. The location and sizing of a DG with reactive power injection have been investigated for loss reduction and cumulative voltage deviation (CVD) enhancement in^22^ to reduce both real and reactive power losses and improve the feeder voltage profile in^23^. The results presented through the arithmetic optimization algorithm (AOA) indicated that specific locations and sizes for multiple DG units delivering active and reactive power can reduce active power loss, voltage deviation, and voltage stability indicator^24^. In^25^, a GWO and PSO approach for selecting the optimal location and sizing of three PV-DGs, that minimize losses and improve the voltage profile has been presented. In^26,27^, the influence of the optimal allocation of different DGs (with different active and reactive power capabilities) into the distribution network has been investigated, which has demonstrated that the DG injecting both real and reactive power has the best technical performance. The best location and size of the renewable resources have been determined by using multi-objective enhanced grey wolf optimizer** (**MOEGWO) improved based on the logistic chaotic mapping integrated with fuzzy decision-making approach, which reduced the operation and emission costs by 23.34% and 34.78%, respectively, and increased the renewable hosting capacity by 7.62%^28^. The result of^29^ indicated that the number of DGs, their location, active power, and reactive power of each DG should be optimized to reach a significant loss reduction. Although many distributed generators are equipped with power electronic devices and modern nonlinear loads that inject harmonics into the network, the aforementioned DG’s allocation problem formulations have not considered harmonic pollution. In^30^, the nonlinear load harmonics injection has been considered for the optimal DG’s size, while the harmonics of the DGs are ignored, resulting in a confusing conclusion such that PV penetration reduces the harmonics, which contradicts the hosting capacity studies in^31^.

The total harmonic distortion due to penetration of nonlinear load and DG using a passive filter has been performed in^32,33^, but the DG location has not been involved in the problem formulation. In^31,34^, passive filters have been designed to mitigate harmonic pollution in single-bus distribution systems, which have neglected voltage profile and system losses. In^35^, the fuel cell and passive filter planning problem has been solved to limit the harmonic pollution, minimize the filter cost, and maximize the benefits in terms of active losses reduction, reactive losses reduction, and power exchange with the grid. However, several other issues have not been considered, such as the fuel cell’s capability for reactive power injection and the expected load growth. In addition, PPF require an additional cost and may be subjected to excessive overheating and system resonance^36,37^. In^38^, the harmonics of DG and nonlinear load have been considered during DG sizing and placement to minimize the total losses and keep harmonics within limits. In^39^, the allocation of three DGs (wind turbine, PV, and fuel cell) in the distribution network has been done to minimize installation cost, energy losses, and emission cost considering load growth. In^38,39^, the dispatch capability of DG in both active and reactive power has not been considered. NSGA-II and Fuzzy have been utilized for distribution system reconfiguration (DSR) to improve the PV hosting capacity, enhance the voltage quality, and minimize the grid loss, including the harmonic of nonlinear loads^40^. From this literature, for optimal planning of the DG, the problem formulation shall consider the harmonic injection from DG and nonlinear loads, the expected load growth to enhance the system sustainability, the reactive power dispatch capability of the DG, which can be used to regulate the voltage and harmonics within limits, and the active power dispatch capability of the DG to provide optimal operation during different loading conditions.

This paper proposes a framework for minimizing the power losses, enhancing the voltage profile, and considering the harmonic pollution from both the nonlinear loads and the DG to follow the IEEE-519 standards for voltage distortion. The proposed framework comprises three stages. The first stage establishes the optimal size and location of the DG for base load operation. If the levels of nonlinear load grow, the amount of harmonic pollution may violate the limits. Therefore, the second stage redesigns the DG size, considering the DG’s tie bus from the first stage, to mitigate the harmonics and optimize the system during high level of nonlinear loads penetration. Upon determining the size and location of the DG from the two stages, the DG rating and connection bus are determined. Eventually, during run time, the third stage optimizes the DG generation to optimize the system operation based on the active and reactive power dispatch capability of the DG. All three stages are optimized employing the genetic algorithm (GA), which has recently been utilized with success in many numerical and combinatorial optimization problems. Two DG’s harmonic spectrums are investigated; one is based on data obtained from^39^, while the other is based on recently issued IEEE 1547-2018 for permissible DG distortions limits^41^. The introduced methodology involves both fundamental and harmonic power flow using MATPOWER^42^. A comparison of literature work and proposed framework is summarized in Table 1.

**Table 1:** Comparison of literature and proposed methodology.

Year	Refs.	Optimization technique	Objective	Active power support of DG	Reactive power support of DG	Load variation	Nonlinear load	Harmonics of DG	Description
2023	^3^	MOAGWO	HC and APL	✓	–	✓	–	–	MOAGWO has been utilized to maximize the hosting capacity and minimize the total energy losses
2018	^9^	NN-ABC	APL and VSI	✓	–	–	–	–	The ABC has been exploited to determine the optimal sizing of fuel cells to minimizes the real power losses and improves the voltage stability index
2021	^10^	GA	APL and VD	✓	–	–	–	–	The position and size of DG unit issue has been solved by considering using genetic algorithm GA for reducing power losses and voltage deviation
2021	^12^	SPBO	APL, TVD, and VSI	✓	–	–	–	–	SPBO has been suggested to solve the multi-objective DG allocation problem considering the power loss, the total voltage deviation, and the voltage stability index
2024	^13^	ECOA	Cost	✓	–	–	–	–	ECOA has been applied to optimize renewable energy sources and pumped-storage hydroelectric units for cost reduction
2023	^15^	SDO	Cost, EM, VD, or APL	✓	–	–	–	–	SDO has been used for optimal power flow to minimize both fuel cost and real power losses
2021	^18^	MFO	AEL, VD, grid power, and demand deviation	✓	–	✓	–	–	MFO has been used for optimal integration of wind turbines, PV, and fuel cells, while considering objectives that include the minimization of annual energy loss, node voltage deviation, and load demand fluctuation in the network
2021	^20^	MMPO	APL and VSI	✓	–	✓	–	–	MMPO has been used to for DGs and simultaneous distribution network reconfiguration at light, nominal, and heavy loading levels for loss and voltage enhancement
2015	^22^	BSOA	APL and CVD	✓	✓	–	–	–	BSOA has been employed for optimal location and size of DGs to minimize power losses and cumulative voltage deviation
2022	^23^	IPSO	APL, RPL, and VD	✓	✓	–	–	–	IPSO has been utilized for optimal allocation and size of the DG units to minimize the active and reactive power losses reduction and enhancement the voltage profile
2023	^25^	GWO-PSO	APL, RPL, and VD	✓	✓	–	–	–	A hybrid GWO and PSO have been utilized to determine the optimal placement and DG size for active and reactive power loss and voltage profile improvement
2024	^26^	GAAOA	APL and VD	✓	–	✓	–	–	A hybrid GAAOA has been introduced to allocate and size of wind turbine, fuel cell, and PV for active power loss minimization and voltage profile enhancement
2023	^27^	BWO	Cost, APL, and EM	✓	✓	–	–	–	BWO has been used for optimal DG allocation in the distribution network to maximize the financial techno-economic, and environmental benefits of the grid
2024	^28^	MOEGWO	HC, Cost, and Emission	✓	–	✓	–	–	MOEGWO integrated with a fuzzy decision-making approach has been used to determine the best location and size of the renewable resources to minimize the operation and emission costs while maximizing the renewable hosting capacity
2022	^30^	PSO	ALI and VSI	✓	–	✓	✓	–	A PSO has been designed to find optimal placement and DG size in IEEE-33-bus and IEEE-34-bus radial distribution systems while considering nonlinear load harmonic injection, although it does not take into account the harmonics injected due to DG
2021	^31^	MOFA	HC	✓	–	–	✓	✓	MOFA has been applied to the optimal design of a third-order damped filter and the size of the distributed generation for enhancement of power quality-constrained hosting capacity
2023	^38^	GA	APL	✓	–	–	✓	✓	GA has been used for optimal sizing and location of DG in an unbalanced network considering the total system losses and harmonics from nonlinear loads and DG
2022	^40^	NSGA-II + Fuzzy	HC, VD or AEL	✓	–	✓	✓	✓	NSGA-II + Fuzzy has been applied to enhance the hosting capacity of radial distribution considering the harmonic pollution from nonlinear loads and DG
**Proposed**		GA	APL	✓	✓	✓	✓	✓	GA is proposed for Optimal sizing and allocation of the hydrogen-based DG (fuel cell) with active and reactive power capability to minimize active power losses, enhance the voltage profile, and limit the individual and total harmonic distortion results from both nonlinear loads and inverter-based DG

*AEL* Annual Energy Losses; *ALI* Active power losses index, *APL* Active power losses, *HC* Hosting capacity, *VD* Voltage deviation, *VSI* Voltage stability index, *RPL* Reactive power Losses.

The major contributions of the paper are summarized as follows:

1. The proposed framework optimizes the DG sizing and location for minimum power losses and to limit the harmonic pollution from both the nonlinear load and DG according to IEEE standards. Hence, the proposed methodology avoids the necessity of harmonic mitigation techniques such as passive or active filters. In addition, avoid passive filter challenges such as resonance and/or overheating due to excessive harmonic absorption.
2. The proposed methodology considers the growth of the loads above the base case with a high penetration of modern nonlinear loads. Accordingly, the proposed methodology enhances the system’s sustainability.
3. The proposed methodology utilizes the active and reactive power dispatch capability of the fuel cell to optimize the system during runtime operation, such as operation at variable load throughout the day. During light loading intervals, the DG injects more reactive power to enhance the voltage profile, while at higher loading, the DG injects more active power with less reactive power to reduce system losses and avoid harmonic violation.

The rest of the article is organized as follows: Section “Problem formulation” illustrates the problem formulation for optimal DG size and location, illustrating system losses as an objective function and various system operating constraints. Section “Power system modeling for harmonic analysis” presents the different-network components model for both harmonic power flow and harmonic analysis. Section “Proposed framework for optimal DG incorporation” elaborates on the proposed multistage framework and optimization process. Section “Optimal DG incorporation into IEEE 33-bus system” applies the proposed methodology on IEEE 33-bus system and investigates the IEEE 33-bus system performance without and with DG for daily load variation with expected load growth. Section “Comparison of the proposed methodology with reported works” provides a comparative investigation of the IEEE 33-bus system performance, utilizing the suggested methodology and some existing literature. Section “System performance according to IEEE 1547–2018” illustrates the DG sizing and allocation considering current distortion requirements by IEEE 1547–2018. Section “Computational time analysis” discusses the computational time of the proposed framework during different stages. Eventually, Section “Conclusion and future research” provides concluding remarks on the presented work and future research directions.

## Problem formulation

This paper works for optimum sizing and allocation of the distributed generation within the distribution system while maintaining safe operation limits for loads and equipment. The problem is described in terms of the objective function and a set of operational constraints.

### Objective functions

According to the literature, minimum power generation losses is utilized in refs.^3,12,17^ while minimum cost is utilized in refs.^13,15,16^. When all resources have the same generation cost function, then the minimum cost and minimum losses provide similar results. Otherwise, minimum losses provide better utilization of the energy, while minimum cost provides better operation cost. For better utilization of the available energy resources, the proposed strategy’s goal is minimizing the total power loss (${P}_{Total\_loss}$) of the system, as shown in Eq. (1). The power losses of the individual branch (${P}_{loss,k}$) are computed using Eq. (2). These losses comprise fundamental power losses (${P}_{loss,k}^{h=1}$) and losses due to harmonic injection (${P}_{loss,k}^{h}$). Where $h$ is the harmonic order. As a result, the total losses (${P}_{Total\_loss}$) within the network are determined from the summation of all branches' losses as given by Eq. (3). Where: m is the total number of branches. The fundamental power losses are computed from the fundamental current as given by Eqs. (4), while the individual harmonic power losses are determined using Eqs. (5). Where $\left|{I}_{k}\right|, {R}_{k}$ are the magnitude of current flows through branch $k$ and the resistance of branch $k$,respectively.

1 $$OF:=\text{min}{P}_{Total\_loss}$$

2 $${P}_{loss,k}={P}_{loss,k}^{h=1}+\sum_{h=2}^{h\_max}{P}_{loss,k}^{h}$$

3 $${P}_{Total\_loss}={\sum }_{k=1}^{m}{P}_{loss,k}={\sum }_{k=1}^{m}{P}_{loss,k}^{h=1}+\sum_{k=1}^{m}{\sum }_{h=2}^{h\_max}{P}_{loss,k}^{h}$$

4 $${P}_{loss,k}^{h=1}=3{\left|{I}_{k}^{h=1}\right|}^{2}{R}_{k}$$

5 $${P}_{loss,k}^{h}=3{\left|{I}_{k}^{h}\right|}^{2}{R}_{k}$$

### Problem constraints

To ensure safe operation of the system, the objective function shall be optimized while the system’s variables do not violate the allowable boundaries. These boundaries appear as constraints in the optimization problem as follows:

#### Bus voltage constraints

For normal operation of installed equipment and loads, the root mean square (RMS) voltage value at all buses shall be maintained within the operation limits^12^. This RMS voltage shall take into account the fundamental and harmonic components as given by Eq. (6). Where ${V}_{i}^{h=1} and {V}_{i}^{h}$ are the RMS values of the fundamental and harmonic voltage components. The voltage constraint is defined using Eq. (7). Where: ${V}^{min}$ and ${V}^{\text{max}}$ are minimum and maximum acceptable voltage levels, which are 0.9 and 1.1, respectively^17^, while ${V}_{i}$ is the bus voltage RMS level.

6 $${V}_{rms\_i}=\sqrt{{\left({V}_{i}^{h=1}\right)}^{2}+\sum_{h=2}^{hmax}{\left({V}_{i}^{h}\right)}^{2}}$$

7 $${V}^{min}\le {V}_{rm{s}_{i}}\le {V}^{\text{max}} \forall i\in \left\{\text{1,2},3,\dots \dots \dots ,n\right\}$$

#### Active and reactive power balance constraints

To keep balance within the system, the generated active power shall equal the demand and total losses inside the system, as given by Eq. (8). Where: ${P}_{g}$,${P}_{Di}$, ${P}_{DG}$ and ${P}_{Losses}$ are the utility grid real power, the demand load at bus $i$, the DG injected real power, and the active power losses, respectively. Similarly, the system shall have a reactive power balance to stabilize the bus voltage. Therefore, the total injected reactive power into the distribution system from the utility (${Q}_{g}$) and the inserted DG (${Q}_{ DG}$) shall equal the total reactive power of the demand (${Q}_{Di}$) and network losses (${Q}_{loss,k}$) as given by Eq. (9). The branch reactive power loss is the sum of the fundamental reactive power losses (${Q}_{loss,k}^{h=1}$) and the branch total harmonic reactive power losses (${Q}_{loss,k}^{h}$) which are computed using Eqs. (11) and (12), respectively.

8 $${P}_{g}+{P}_{ DG}={\sum }_{i=1}^{n}{P}_{Di}+{\sum }_{k=1}^{m}{P}_{loss,k}$$

9 $${Q}_{ g}+{Q}_{ DG}={\sum }_{i=1}^{n}{Q}_{Di}+{\sum }_{k=1}^{m}{Q}_{loss,k}$$

10 $${Q}_{loss,k}={Q}_{loss,k}^{h=1}+\sum_{h=2}^{h\_max}{Q}_{loss,k}^{h}$$

11 $${Q}_{loss,k}^{h=1}=3{\left|{I}_{k}^{h=1}\right|}^{2}{X}_{k}$$

12 $${Q}_{loss,k}^{h}=3{\left|{I}_{k}^{h}\right|}^{2}h{X}_{k}$$

#### Branch loading constraints

To avoid excessive loading, the current flow through the branches shall be within the thermal limits as given by Eq. (13). Where ${\left|{I}_{k}\right|}_{max}$ is the maximum allowable current of the branch $k$ without violating the thermal limits.

13 $$\left|{I}_{k}\right|\prec {\left|{I}_{k}\right|}_{max}$$

#### Individual and total harmonics constraints

Inverter based DG and nonlinear loads such as (variable speed drives, computers, LED lighting) inject harmonics into the system. Several power-quality concerns result from harmonics pollution^43^; consequently, for medium voltage network, the maximum individual and total harmonic distortion shall be limited to 3% and 5% as per IEEE-519 respectively^44^. The constraints on the individual and total harmonic distortion at each bus are represented by Eqs. (14) and (15). Where: ${IHD}_{\text{i}}(h)$ and ${IHD}_{max}\left(h\right)$ are individual harmonic distortion at bus $i$ and maximum allowable individual harmonic distortion of harmonic order $h$, respectively. ${THD}_{\text{i}}and{ THD}_{max}$ are the total harmonic distortion at bus $i$ and maximum permissible total harmonic distortion, respectively. The total harmonic distortion is calculated using Eq. (16). The voltage harmonic components are obtained from harmonic power flow, as will be discussed later.

14 $${IHD}_{i}(h)\prec {IHD}_{max}(h)$$

15 $${THD}_{\text{i}}\prec {THD}_{max}$$

16 $${THD}_{i}\%=100\frac{\sqrt{{\sum }_{h=2}^{h\_max}{V}_{i,h}^{2}}}{{V}_{i}^{h=1}}$$

#### Generation limits constraints

For safe unit operation, each generating unit, including the utility, has lower and upper boundaries on the active and reactive power. These restrictions are represented by Eqs. (17) and (18). Where: ${P}_{gen i,min} and {P}_{gen i,max}$ are the minimum and maximum permissible generation of active power at bus $i$, respectively. ${Q}_{gen i,min} and {Q}_{gen i,max}$ are the minimum and maximum generation of reactive power at bus $i$, respectively.

17 $${P}_{gen i,min}\le {P}_{gen i}\le {P}_{gen i,max}$$

18 $${Q}_{gen i,min}\le {Q}_{geni}\le {Q}_{gen i,max}$$

## Power system modeling for harmonic analysis

A large amount of harmonic is injected into the network because of the existence of nonlinear loads and an inverter-based distributed generator. For harmonic power flow and harmonic analysis, the model of each element, including network branches, loads, and generators, is developed in this section.

### Branch modeling

Network branches are represented by a series impedance. For typical harmonic power flow studies, the resistance of the series impedance is assumed to be constant; however, the reactance is proportional to the harmonic order. Therefore, at the harmonic order ($h$), the branch impedance (${z}_{ij}^{h}$) between bus $i and j$ is given by Eq. (19). Where:${x}_{ij}$ is the branch’s reactance at the fundamental frequency. The corresponding admittance (${y}_{ij}^{h}$)for the h-harmonic order is given by Eq. (20).

19 $${z}_{ij}^{h}={r}_{ij}+\text{j }h{x}_{ij}$$

20 $${y}_{ij}^{h}=\frac{1}{{r}_{ij}+j h {x}_{ij}}$$

### Load modeling

The electric load on the network can be classified into linear and nonlinear. At fundamental frequency, the load bus is PQ-bus with an active and a reactive power of the summation of linear and nonlinear load consumption. However, for harmonic power flow (HPF), each load type behaves differently. Linear loads are regarded as shunt admittance, whereas nonlinear loads are treated as harmonic injection current sources^46^.

#### Linear Loads

The linear loads at fundamental frequency are represented by real (${P}_{d,i}$) and reactive (${Q}_{d,i}$) power. The equivalent impedance at the fundamental frequency is given by Eq. (21). Where: ${r}_{d,i}and{x}_{d,i}$ are the equivalent resistance and fundamental equivalent reactance. Hence, the linear load in the admittance form for the h-harmonic order is expressed by Eq. (22). Where ${y}_{load,i}^{h}$ is the equivalent admittance of a linear load at bus $i$ for harmonic order $h$.

21 $${r}_{d,i}+ \text{j}{x}_{d,i}=\frac{{V}_{i}^{2}}{{P}_{d,i}-j{Q}_{d,i}}$$

22 $${y}_{load,i}^{h}=\frac{1}{{r}_{d,i}+\text{j} h {x}_{d,i}}$$

#### Nonlinear Load

For HPF, the nonlinear loads are treated as current injectors. The amplitude and phase shift of the current source depend on the fundamental current and harmonic order. The fundamental demand current is determined from the fundamental power flow analysis as expressed by Eq. (23). Where:${P}_{nl,i}and{Q}_{nl,i}$ are the nominal real and reactive power of the nonlinear load at bus $i$, and ${I}_{nlj,i}^{h=1}$ is the fundamental injected current of the nonlinear load.

23 $${I}_{nlj,i}^{h=1}= {\left(\frac{{P}_{nl,i}+{Q}_{nl,i}}{{V}_{i}}\right)}^{*}$$

The injected current for each harmonic order is determined from the harmonic spectrum as given by Eqs. (24) and (25). Where: $\left|{I}_{nlj,i}^{h}\right|$ and $\left|{I}_{nlj,i}^{h=1}\right|$ are the RMS of the injected current at harmonic order $h,$ and at fundamental frequency, respectively. Where: ${I}_{spectrum}^{h}$ and ${\theta }_{spectrum}^{h}$ represent the harmonic spectrum. ${\theta }_{nlj,i}^{h=1}and{\theta }_{nlj,i}^{h}$ are the phase shift of the injected current at the fundamental frequency and at the harmonic order $h$, respectively. A typical harmonic spectrum for nonlinear loads is provided in Table 2^42^.

**Table 2:** Typical Nonlinear Load Harmonic Spectrum^42^.

Harmonic Order	Typical harmonic spectrum	
	${I}_{spectrum}^{h}\left(\%\right)$	${\theta }_{spectrum}^{h}\left(\%\right)$
1	100	0
5	18.24	−55.68
7	11.9	−84.11
11	5.73	−143.56
13	4.01	−175.58
17	1.93	111.39
19	1.39	68.3
23	0.94	−24.61
25	0.86	−67.64
29	0.71	−145.46
31	0.62	176.83

24 $$\left|{I}_{nlj,i}^{h}\right|= {I}_{spectrum}^{h}\left|{I}_{nlj,i}^{h=1}\right|$$

25 $${\theta }_{nlj,i}^{h}={\theta }_{spectrum}^{h}+{h\theta }_{nlj,i}^{h=1}$$

### DG unit modeling

During fundamental power flow analysis, the DG is represented by PQ, which injects active and reactive power. For HPF, the fuel cell with inverter is represented by a current source injecting harmonics into the network. Table 3 presents the typical harmonic spectrum of an inverter-based DG^39^. The fundamental injected current (${I}_{DG,i}^{h=1}$) is computed from the fundamental power flow using Eq. (26). The injected DG current at harmonic order is obtained utilizing Eqs. (27) and (28). Where ${I}_{D{G}_{spectrum}}^{h}$ and ${\theta }_{DG\_spectrum}^{h}$ are the DG harmonic spectrum magnitude and phase shift.${\theta }_{DG,i}^{h=1}$ is the fundamental current phase shift.

**Table 3:** Typical Harmonic Spectrum of Inverter-Based DG Unit^39^.

Harmonic Order	${I}_{spectrum}^{h}\left(\%\right)$
1	100
5	19.14
7	13.09
11	7.58
13	5.86
17	3.79
19	3.29
23	2.41
25	2.26
29	1.93
31	1.81

26 $${I}_{DG,i}^{h=1}= {\left(\frac{{P}_{DG,i}+{Q}_{DG,i}}{{V}_{i}}\right)}^{*}$$

27 $$\left|{I}_{DG,i}^{h}\right|= {I}_{DG\_spectrum}^{h}\left|{I}_{DG,i}^{h=1}\right|$$

28 $${\theta }_{DG,i}^{h}={\theta }_{DG\_spectrum}^{h}+{h\theta }_{DG,i}^{h=1}$$

### Grid modeling

The utility grid works as a slack bus to compensate for the mismatch between demand and DG generation. Several studies consider the utility to be an ideal source with zero internal impedance. However, in practical, the grid has an internal impedance (${Z}_{grid}$) which plays a vital role in limiting the short circuit current on medium voltage^45^. Therefore, the equivalent grid impedance is determined from the short circuit level (${S}_{sc}$) and nominal network voltage (${V}_{n}$) at the point of connection, as given by Eq. (29). According to Schneider recommendations, the grid reactance and resistance are determined from Eqs. (30) and (31), respectively^45^. The equivalent grid impedance during harmonic analysis is given by Eq. (32). The summary of the equivalent models for various elements in harmonic analysis is provided in Table 4.

**Table 4:** Equivalent models for harmonic analysis.

Element	Branch	Slack (Grid)	Linear load	Nonlinear load or DG
Equivalent Circuit				

29 $${Z}_{grid}= \frac{{V}_{n}^{2}}{{S}_{sc}}$$

30 $${X}_{grid}\approx 0.98{Z}_{grid}$$

31 $${R}_{grid}=\sqrt{{\left({Z}_{grid}\right)}^{2.}-{\left({X}_{grid}\right)}^{2.}}$$

32 $${Z}_{grid}^{h}={R}_{grid}+Jh{X}_{grid}$$

### Bus voltage calculation

The bus voltage at the harmonic order ($h$) is determined using the equivalent Z-bus matrix, and the injected current into the bus at the corresponding harmonic order is given by Eq. (33). Where : ${\text{V}}_{\text{bus}}^{\text{h}}$ is the column vector of bus voltages, ${\text{I}}_{\text{bus}}^{\text{h}}$ is the column vector of bus injection currents, ${\text{Z}}_{\text{bus}}^{\text{h}}$ is the impedance matrix of the distribution system.

33 $${V}_{bus}^{h}={Z}_{bus}^{h}{I}_{bus}^{h}$$

## Proposed framework for optimal DG incorporation

**Figure 1:**
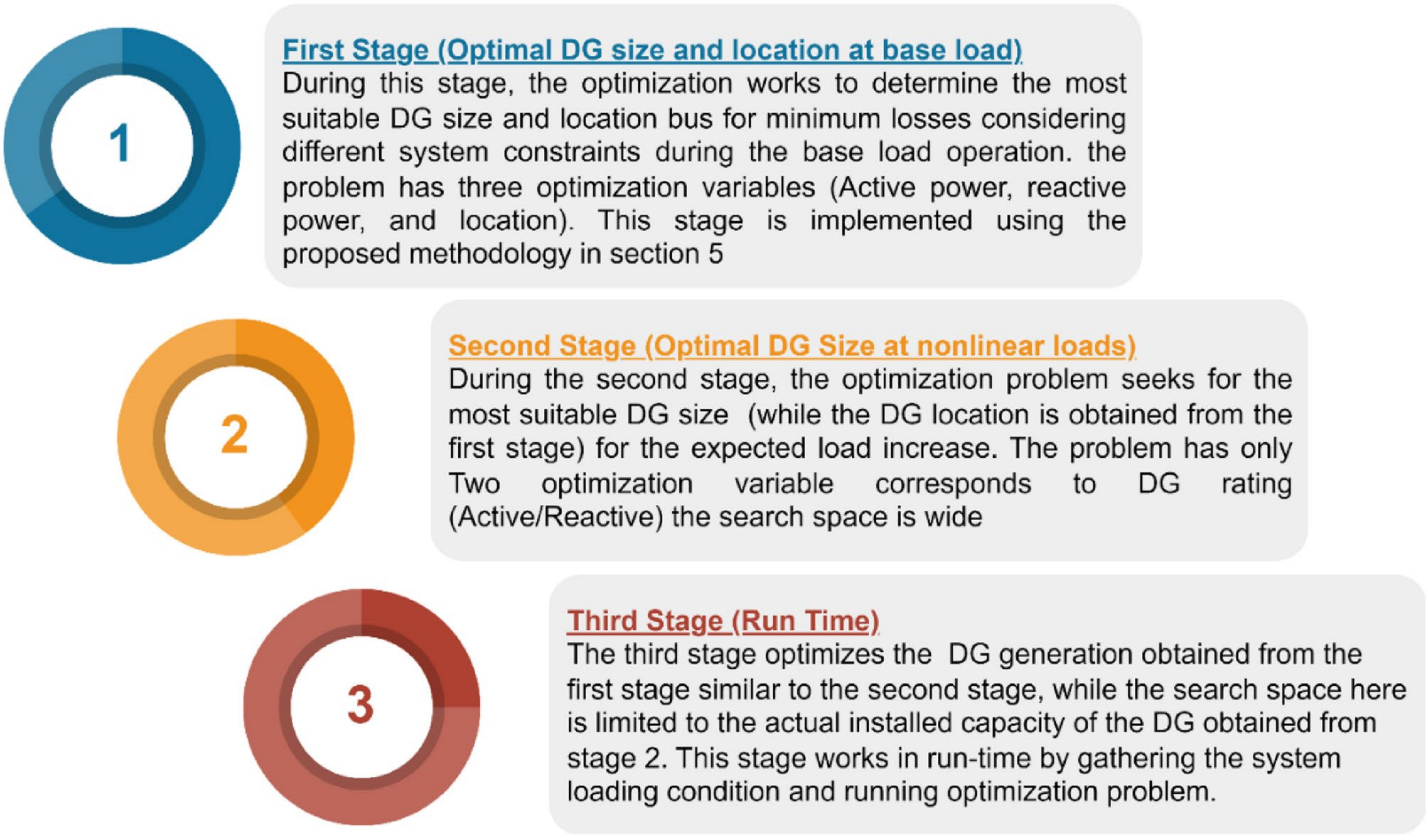
Proposed multi-stage framework for DG incorporation.

The size of DG at a particular operating condition provides optimal operation at this condition. While at load change, the system may exhibit higher losses, and some constraints are violated, especially the harmonic content in the presence of a nonlinear load. Therefore, the proposed framework determines the DG location and size to provide optimal operation of a wide range of nonlinear load variations utilizing the active and reactive power dispatch capability of the DG. The proposed methodology involves three stages, as shown in Fig. 1. The first stage optimizes the DG size and location, considering the base load for minimum operation losses considering the system harmonics, voltage deviation, and generation constraints. The second stage optimizes the DG size, while the DG location is maintained at a selected bus by the first stage to mitigate the harmonics and optimize the system losses during high levels of nonlinear load penetration. After finalizing the second stage, the DG size is selected to be able to optimize the system at different loading conditions (linear and nonlinear). Eventually, during run time, the third stage utilizes the power dispatch capability of the DG to specify the optimal DG generation for minimizing system losses and maintaining the system variables within allowable boundaries according to the actual loading condition of the system. The third stage is limited by the available DG size and tie location, which are obtained from stages 1 and 2.

### Schematic diagram of the optimization process

**Figure 2:**
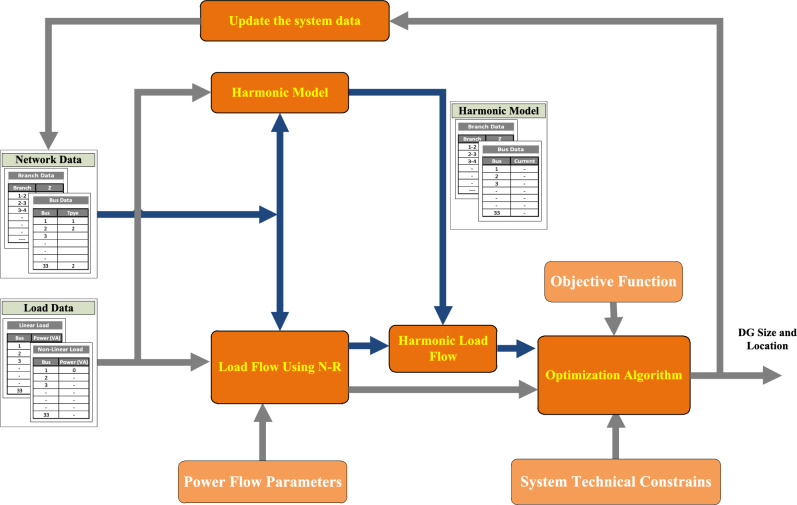
Proposed methodology for DG sizing and allocation.

The sizing and allocation of DG for optimizing the system losses without violating the harmonic constraints involves fundamental power flow, harmonic power flow, and optimization techniques, as shown in Fig. 2. The methodology receives the network data (branches, buses, etc.) and loads data (linear and nonlinear) as input data. Then Newton–Raphson is used to perform fundamental power flows. According to the results of the fundamental power flow and harmonic model of the network (which are developed in Section “Power system modeling for harmonic analysis”), harmonic power flow is performed. From both the fundamental power flow and harmonic flow results, the optimization technique verifies the system constraints and suggests the DG size and location to minimize the system losses. According to the suggested DG size and location, the system model is updated, and the optimization process is repeated until the stopping condition is satisfied.

### Optimization technique for various stages

**Figure 3:**
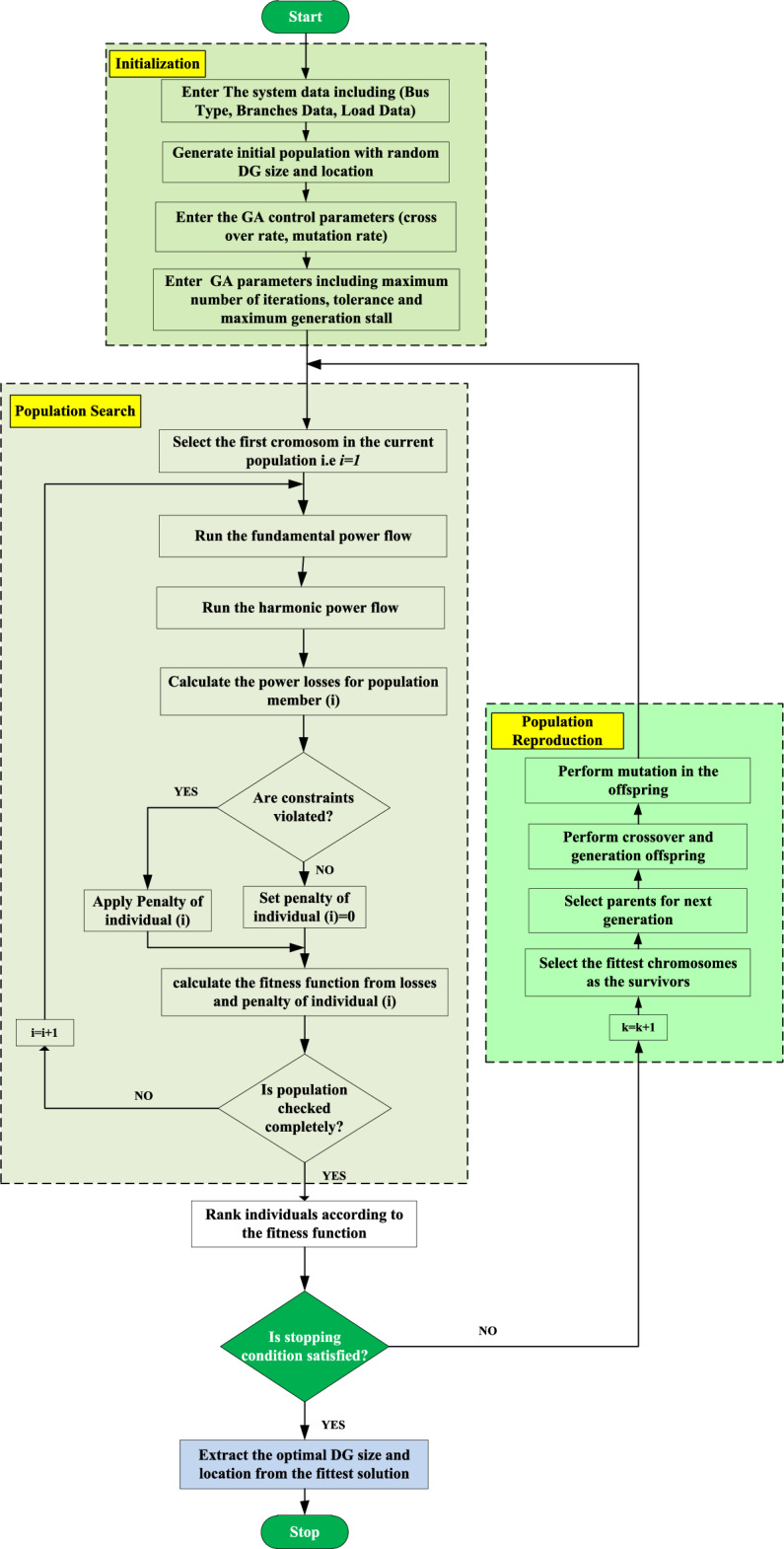
Flowchart of genetic algorithm for optimal DG size and location.

There are many optimization algorithms that can be applied, such as evolutionary programming (EP), cuckoo search, particle swarm optimization (PSO), and genetic algorithms (GA)^38^. All the aforementioned techniques are derivative-free and can deal with linear and nonlinear problems. Compared to traditional methods, GA is faster, more efficient, suitable for continuous and discrete optimization problems, and has the capability of working with mixed-integer problems^10^. The GA imitates the process of natural selection, in which the best-fitting solutions are selected as survivors. These survivors are called parents since they are used to produce offspring for the next generation. The process of offspring production is implemented through two processes: The first is a crossover, in which genes from different parents are swapped to reproduce a new individual. The second process is a mutation, in which a mutation appears in the new generation to enhance optimization diversity. The processes of mutation and crossover are controlled by mutation rates and crossover rates, respectively. The flowchart of the proposed optimization methodology using GA is shown in Fig. 3. The optimization process starts with entering line data, bus data, system constraints, and GA data. After that, the optimization process generates a random initial population in solution space with a randomly selected size and location of DGs. For all solutions, calculate `the distribution system power losses using the load flow process and check for constraints violations. Then, after determining the best solution, calculate the minimum losses for this iteration. This process is repeated for all the iterations. When the stooping condition is satisfied, extract the optimal position and capacity of the DG. The optimal DG size and location are required in the first stage of the suggested framework. The second-stage optimization process has two optimization variables corresponding to DG’s size (active and reactive power), while DG location is extracted from the first-stage results. The parameters of GA are given in Table 5.

**Table 5:** Genetic Algorithm setting Parameters for different stages.

	First Stage	Second Stage	Third Stage
Operating condition	Operation at linear base load	Operation at high level of nonlinear load	Run time operation
Optimization Variables	Rated DG active and reactive power DG location	Rated DG active and reactive power	DG generation active and reactive power
System constraints	Constraints of subsection “Problem Constraints”	Constraints of subsection “Problem Constraints” DG tie bus of the first stage	Constraints of subsection “Problem Constraints” DG tie bus of the first stage DG rated active and reactive power of the second stage
Number of variables	3	2	2
Population Size	50	50	50
Generations	200	200	200
Crossover rate	0.8	0.8	0.8
Mutation rate	0.2	0.2	0.2

## Optimal DG incorporation into IEEE 33-bus system

**Figure 4:**
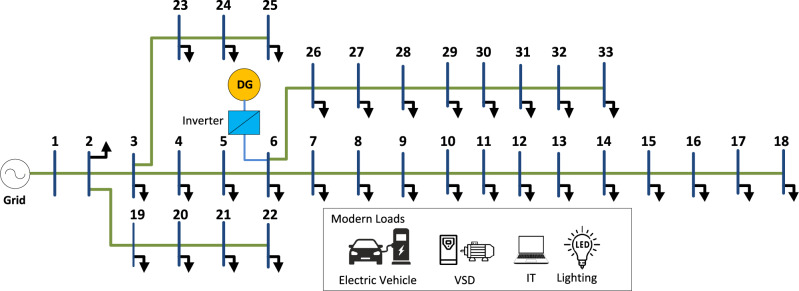
Modified IEEE-33-bus radial distribution network.

In this section, the proposed framework is applied to optimally incorporate DG into the IEEE 33-bus system as shown in Fig. 4 to minimize the real power losses, maintain the bus voltage, and keep harmonic distortion within permissible limits. The system’s peak active power demand is 3.7 MW and its peak reactive demand is 2.3 Mvar, with a base power of 10 MVA and a rated voltage of 12.08 kV. The system data showing the bus data, branch data, and generator data is provided in^3^. The system losses, voltage, and harmonics are evaluated with and without DG penetration for base load operation and nonlinear load change by ± 50%, and load variation continues for 24 h.

### IEEE 33-bus system performance during base load without and with DG (First stage)

**Figure 5:**
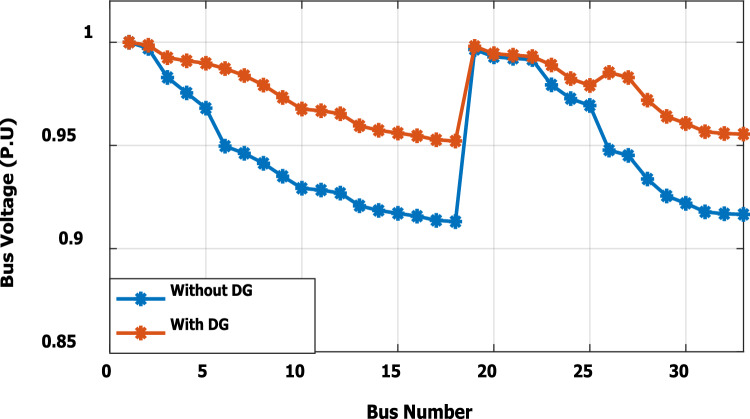
Voltage profile for base case without DG and with DG at bus 6.

In the base case, the system is working under the normal peak load of the distribution network. Without DG penetration, the minimum bus voltage is 0.913 p.u. at bus 18, because this bus is located at the dead end of the network. The system has total active and reactive losses of 203 kW and 140 kvar, respectively. The proposed methodology suggests that the optimal DG is allocated at bus no. 6, with a power injection capacity of 1.799 MW and 1.288Mvar. Because of the ability of DG to provide reactive power, the voltage profile has been improved as compared with base case results without DG, as presented in Fig. 5. The dead-end voltage at bus 18 is improved to 0.952 instead of 0.913 p.u.

**Figure 6:**
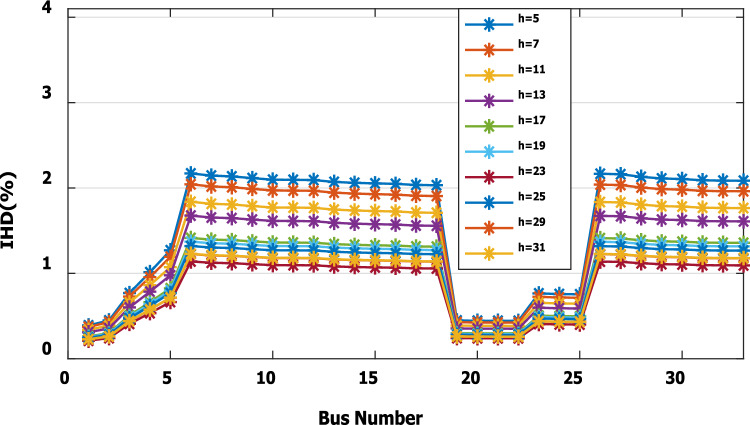
IHD for the base case with DG at bus 6.

**Figure 7:**
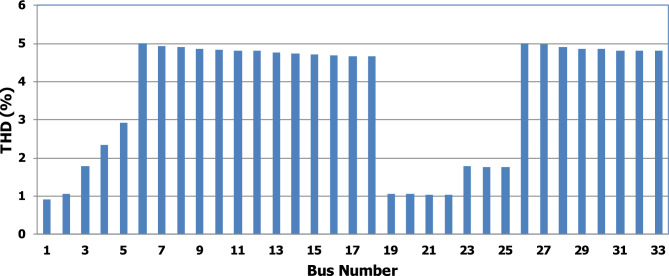
THD for the base case with DG at bus 6.

For base load and DG installation at bus 6, the loads are considered linear loads. Hence, the only source of harmonic injection is the DG. The maximum individual harmonics (IHD) and the total voltage harmonic distortion (THD) at all buses within the network are less than 3% and 5%, as shown in Fig. 6 and Fig. 7, respectively. The IHD and THD attained 2.17% and 5%, respectively, which do not exceed the limitation set by IEEE Standard 519. Hence, for base load, the DG installation at bus 6 minimizes the losses, maintains the voltage within the limits, and maintains the harmonics within the permissible range. Moreover, with optimal DG allocation, the real power loss is reduced from 203 kW to 76.282 kW and the reactive power loss is reduced from 140kvar to 74.401 kvar as shown in Table 9.

### Optimal DG sizing for IEEE 33-bus system (second stage)

At nonlinear load change, the voltage drops, and the harmonics contents are changed. Hence, the optimal DG size changes. The second stage of the proposed framework searches for the optimal DG rating (the DG connection bus is the same as the first stage suggestion) to minimize the system losses while maintaining the harmonics within acceptable limits at increasing or decreasing the nonlinear load. This stage is performed in two operating scenarios which are load increases by 50% and decreases by 50% in the presence of nonlinear loads.

#### Optimal DG rating with 50% nonlinear load penetration for IEEE 33-bus system

**Figure 8:**
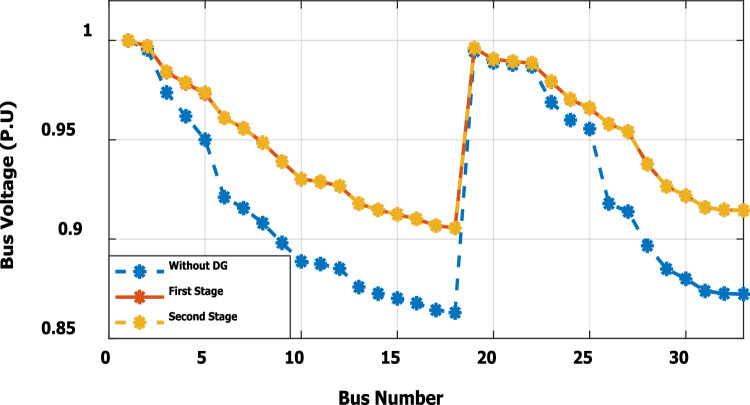
Voltage profile at 50% increase of nonlinear loads without and with DG.

**Figure 9:**
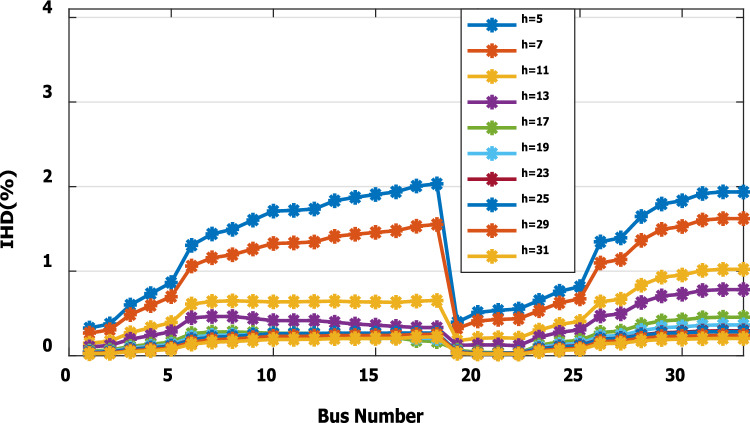
IHD at nonlinear load increased by 50% without DG.

**Figure 10:**
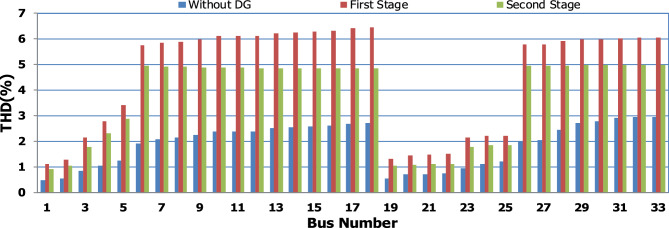
THD at nonlinear load increased by 50% without and with DG.

**Figure 11:**
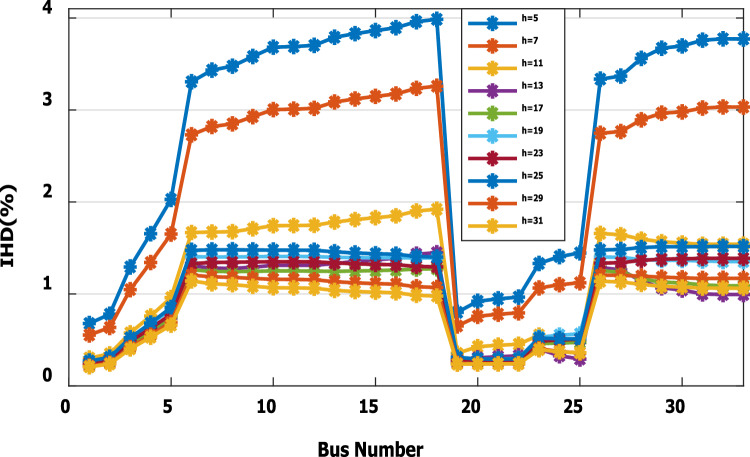
IHD at nonlinear load increased by 50% with DG of first stage.

**Figure 12:**
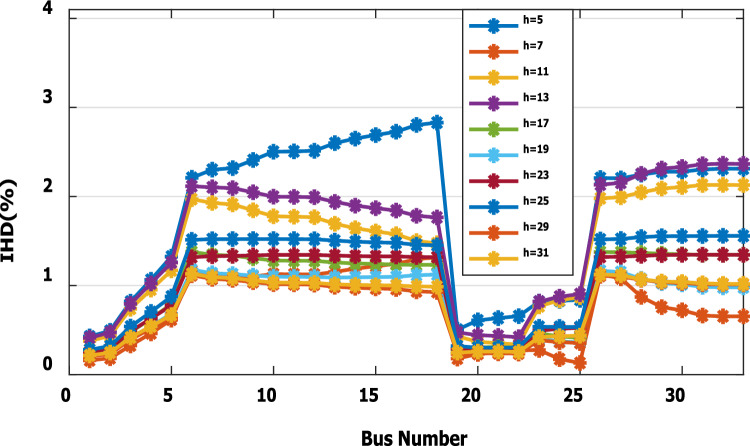
IHD at nonlinear load increased by 50% with DG of second stage.

The nonlinear load level is increased by 50% of the total load at each bus. Without DG, the minimum bus voltage at bus 18 is 0.863 p.u., which is outside the acceptable limits as shown in Fig. 8, but the individual harmonics and total voltage harmonic distortion at all buses within the network are less than 3% and 5%, as shown in Figs. 9 and 10, respectively. With DG placement at bus 6 with the same size of the first stage (1.799 MW and 1.288 Mvar), the voltage at the 18th bus is improved to 0.906 p.u., while the 5^th^ harmonic exceeds the permissible 3% at buses 6–18 and 26–33, as shown in Fig. 11. In addition, the THD exceeds the permissible 5% at buses 6–18 and 26–33, as presented in Fig. 10. The maximum individual harmonic distortion (IHD) and total voltage harmonic distortion (THD) attain 3.986% and 6.448% (at bus 18), respectively. From these results, even though the DG enhanced the voltage deviation to be within limits, harmonic distortion exceeded the limitation set by IEEE Standard 519. Running the second stage of the proposed framework with the same DG at bus 6, the optimal rating of the DG is 2.097 MW and 0.829 Mvar. Compared to without DG, as shown in Fig. 8, the minimum voltage is improved from 0.863 to 0.906 p.u. Compared to the DG of the first stage, the new DG’s rating maintains the maximum individual and THD within the permissible limits, as shown in Fig. 10 and Fig. 12.

Table 6 provides a comparison between the performance of the system with the DG rating of stage 1 and the DG rating suggested by stage 2 at a load increase by 50%. Compared to without DG, DGs of stages 1 and 2 enhance the maximum voltage deviation within the network from 13.7 to 9.4 p.u., which maintains the deviation within the permissible limits. While, with DG of stage 1, the system's maximum individual and total harmonic distortions are 3.986% and 6.448%, respectively, which violate the permissible limits. On contract with DG of stage 2, the maximum IHD and the maximum THD have a value of 2.83% and 4.999%, respectively. Moreover, with a DG rating of stage 2, the real and reactive power losses are reduced from 482.583 KW and 327.765 kvar to 227.243 kW and 184.710 kvar, respectively. Hence, the redesign of the DG size is able to minimize the real and reactive losses, enhance the voltage deviation, and maintain the harmonic indicators within the permissible limits. Hence, if the DG is generating the rated capacity at bus 6, the voltage deviation becomes within acceptable limits while the system's total harmonic distortion violates the permissible standard. If the DG generation injects 2.097 MW/0.829 Mvar instead of 1.799 MW/1.288 Mvar, the system voltage deviation becomes within the permissible boundaries at 10%. Moreover, the system's maximum individual and total harmonic distortions are 2.83% and 4.99%.

**Table 6:** Performance comparison for IEEE-33 bus system during nonlinear load increased by 50%.

DG size	Load increased by 50%		
	Without DG	1799 + 1288i (stage1)	2097 + 829i (stage 2)
Bus NO	–	6	6
Fundamental ${P}_{losses}(kW)$	481	219	223
Fundamental ${Q}_{losses}(kVAR)$	320	160	160
Harmonic ${P}_{losses}(kW)$	1.583	8.329	4.243
Harmonic ${Q}_{losses}(kVAR)$	7.765	41.702	24.710
Total ${P}_{losses}(kW)$	482.583	227.329	227.243
Total ${Q}_{losses}(kVAR)$	327.765	201.702	184.710
${V}_{min}(P.U)$	0.863	0.906	0.906
${V}_{max}(P.U)$	1	1	1
${\Delta{V}}_{max}\left(\%\right)$	13.7	9.4	9.4
Max IHD (%)	2.033	3.986	2.83
Max THD (%)	2.941	6.448	4.999

#### Optimal DG rating with load is decreased by 50% for IEEE 33-bus system.

**Figure 13:**
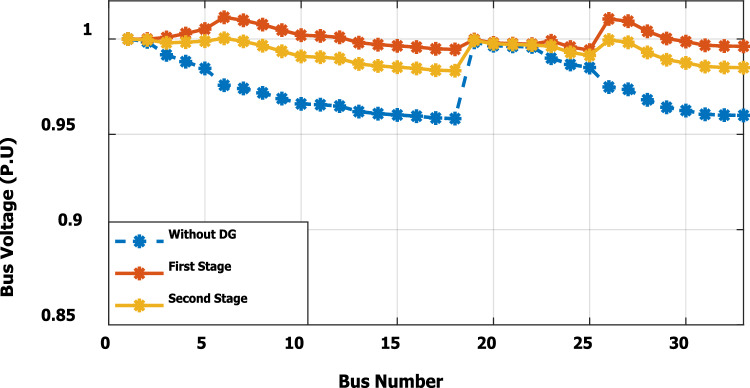
Voltage profile at 50% decrease of loads without and with DG.

**Figure 14:**
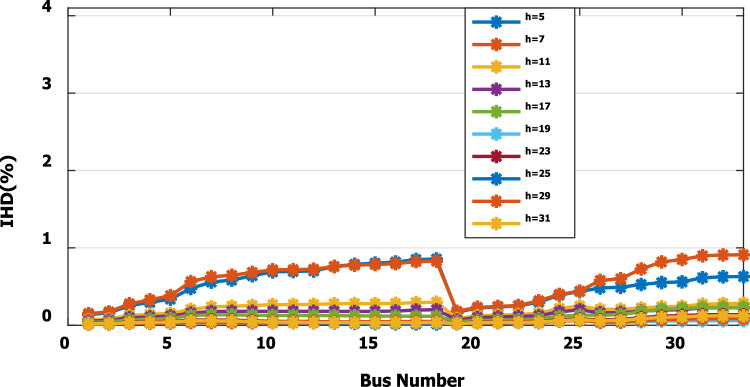
IHD at load decreased by 50% without DG.

**Figure 15:**
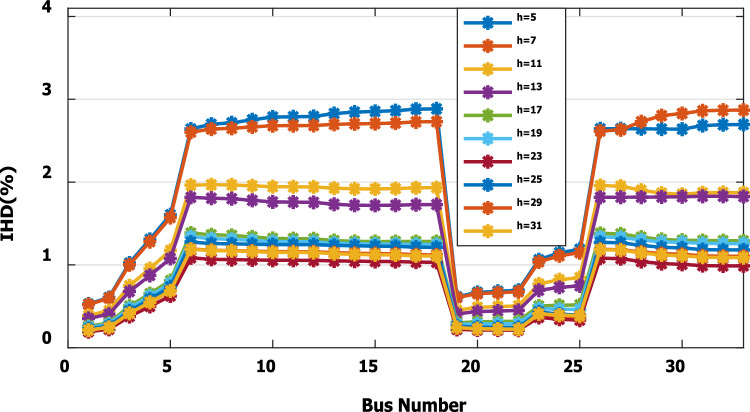
IHD at load decreased by 50% with DG of first stage.

**Figure 16:**
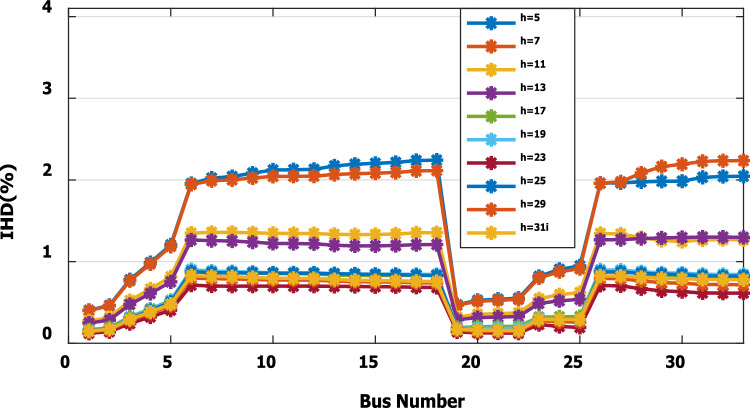
IHD at load decreased by 50% with DG of second stage.

**Figure 17:**
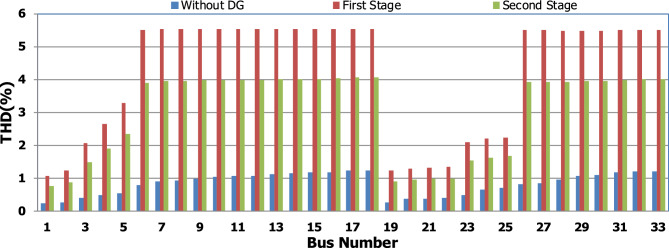
THD at load decreased by 50% without and with DG.

Furthermore, the load is decreased by 50% (loading is 25% of the nonlinear load and 25% of the linear load) of the total distribution system load. The new optimal DG rating is 1.245 MW and 0.856 Mvar. Due to load reduction, the voltage deviation with and without DG is maintained within the limits as shown in Fig. 13, while both the DG sizes of stages 1 and 2 enhance the voltage deviation. Also, the maximum individual harmonic distortion is less than 3% with and without DG, as shown in Fig. 14, Fig. 15, and Fig. 16. On the other hand, the total harmonic distortion exceeds the permissible limits using DG of stage 1 on several buses, as shown in Fig. 17. Table 7 shows, compared to without DG, that with DG of stage 2, the real and reactive power losses are reduced from 47.294 kW to 18.343 kw and from 31.554 to 27.875 kvar, respectively. Moreover, compared to the DG of stage 1, the DG of stage 2 reduces the total harmonic distortion from 5.542 to 4.059% to be within the permissible limits. In addition, there is an enhancement in the individual harmonic distortion. Therefore, the DG of the second stage provides minimum losses and maintains the harmonic distortion within allowable limits.

**Table 7:** Performance comparison for IEEE-33 bus system during load decrease by 50%.

DG size	Load decreased by 50%		
	Without DG	1799 + 1288i (Stage1)	1245 + 856i (Stage 2)
Bus NO	–	6	6
Fundamental ${P}_{losses}(kW)$	47	21	15
Fundamental ${Q}_{losses}(kVAR)$	30	20	10
Harmonic ${P}_{losses}(kW)$	0.294	6.145	3.343
Harmonic ${Q}_{losses}(kVAR)$	1.554	33.469	17.875
Total ${P}_{losses}(kW)$	47.294	27.145	18.343
Total ${Q}_{losses}(kVAR)$	31.554	53.469	27.875
${V}_{min}(P.U)$	0.958	0.994	0.983
${V}_{max}(P.U)$	1	1.011	1
${\Delta{V}}_{max}\left(\%\right)$	4.2	1.1	1.7
Max IHD (%)	0.855	2.885	2.243
Max THD (%)	1.25	5.542	4.059

According to the results of stages 1 and 2, to minimize the system losses and maintain the voltage and harmonics within the acceptable limits for a wide variation of loads ± 50%, the DG generation shall be varied as shown in Table 8. In practical, a specific rating of DG shall be installed, and the generation can be controlled to optimize the system performance while the system constraints are not violated. Hence, to ensure the capability of DG generation, the recommended DG rating is the maximum active power and maximum reactive power capability (2.097 MW, 1.288 Mvar).

**Table 8:** Optimal DG for IEE 33-bus system.

	Base case	load increased by 50%	load decreased by 50%
Optimal DG bus	6	6	6
Optimal real power (MW)	1.799	2.097	1.245
Optimal reactive power(MVAR)	1.288	0.829	0.856
Optimal power factor	0.813	0.93	0.824
Recommended DG size	DG (2.097 MW, 1.288 Mvar) @ bus 6		

### Optimal DG generation with continues load variation along the day (Third stage)

**Figure 18:**
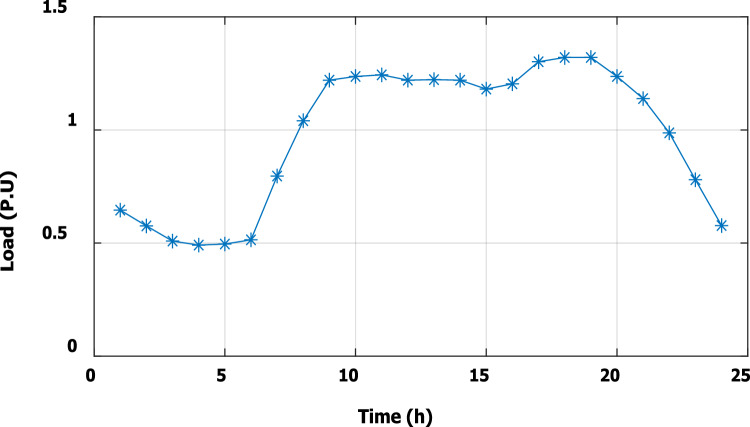
Modified load throughout the day.

**Figure 19:**
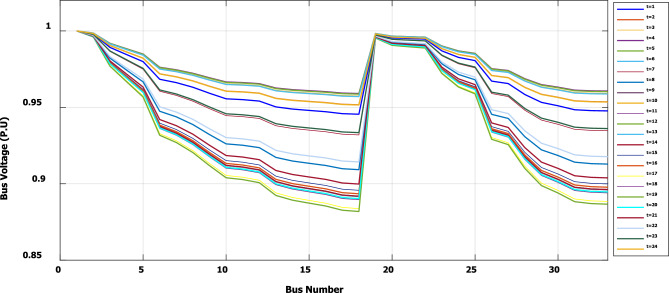
Voltage profile throughout the day without DG.

**Figure 20:**
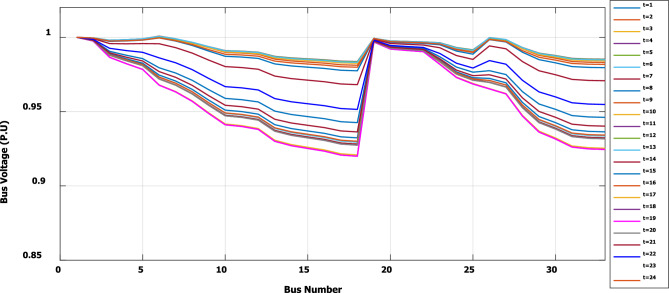
Voltage profile throughout the day with DG.

**Figure 21:**
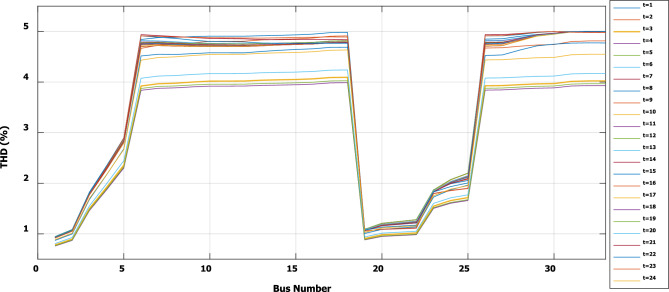
THD throughout the day with DG.

**Figure 22:**
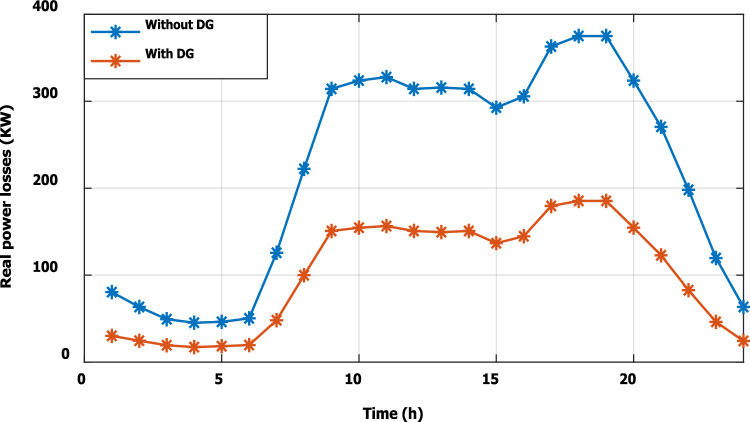
Real power losses throughout the day with and without the DG.

To evaluate the effectiveness of the third stage, all bus linear and nonlinear loads were assumed to vary along the day with a value, which is presented in Fig. 18. During real-time operation, the third stage determines the optimal DG penetration, which is constrained by the rated capacity of the DG obtained from the first and second stages (the rated DG is 2.097 MW and 1.288 Mvar at bus 6). Without DG, the minimum voltage at bus no. 18 becomes less than 0.9 p.u. from time 9 to 20, which is outside the permissible limits as shown in Fig. 19. With DG generation at bus 6, the minimum voltage improved and became within the acceptable limits, as shown in Fig. 20. In addition, the maximum total voltage harmonic distortion (THD) reaches 5% at some intervals due to the DG penetration and the increase of nonlinear loads; however, the THD is still within the limitations set by IEEE Standard 519, as shown in Fig. 21. Moreover, the DG penetration reduces the power losses significantly throughout the day, as shown in Fig. 22. The total energy loss without DG is 5.281 MWh/day, while with DG, the total energy loss is reduced to 2.452 MWh/day, which means 53.6% energy saving.

**Figure 23:**
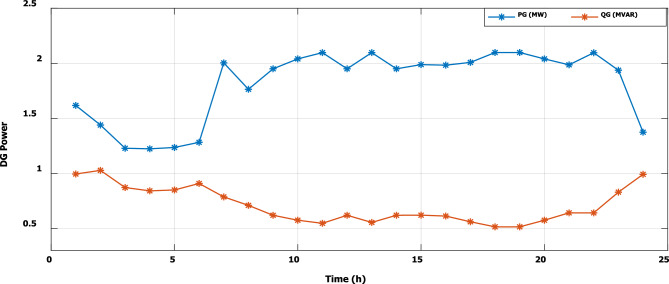
DG generated active and reactive power throughout the day with DG.

Figure 23 shows that, during a high load interval (9:00 o’clock to 20 o’clock), the DG tends to increase its generated active power to minimize the real power losses based on local generation, while to avoid excessive harmonic injection, the DG tends to reduce the generated reactive power.

## Comparison of the proposed methodology with reported works

**Figure 24:**
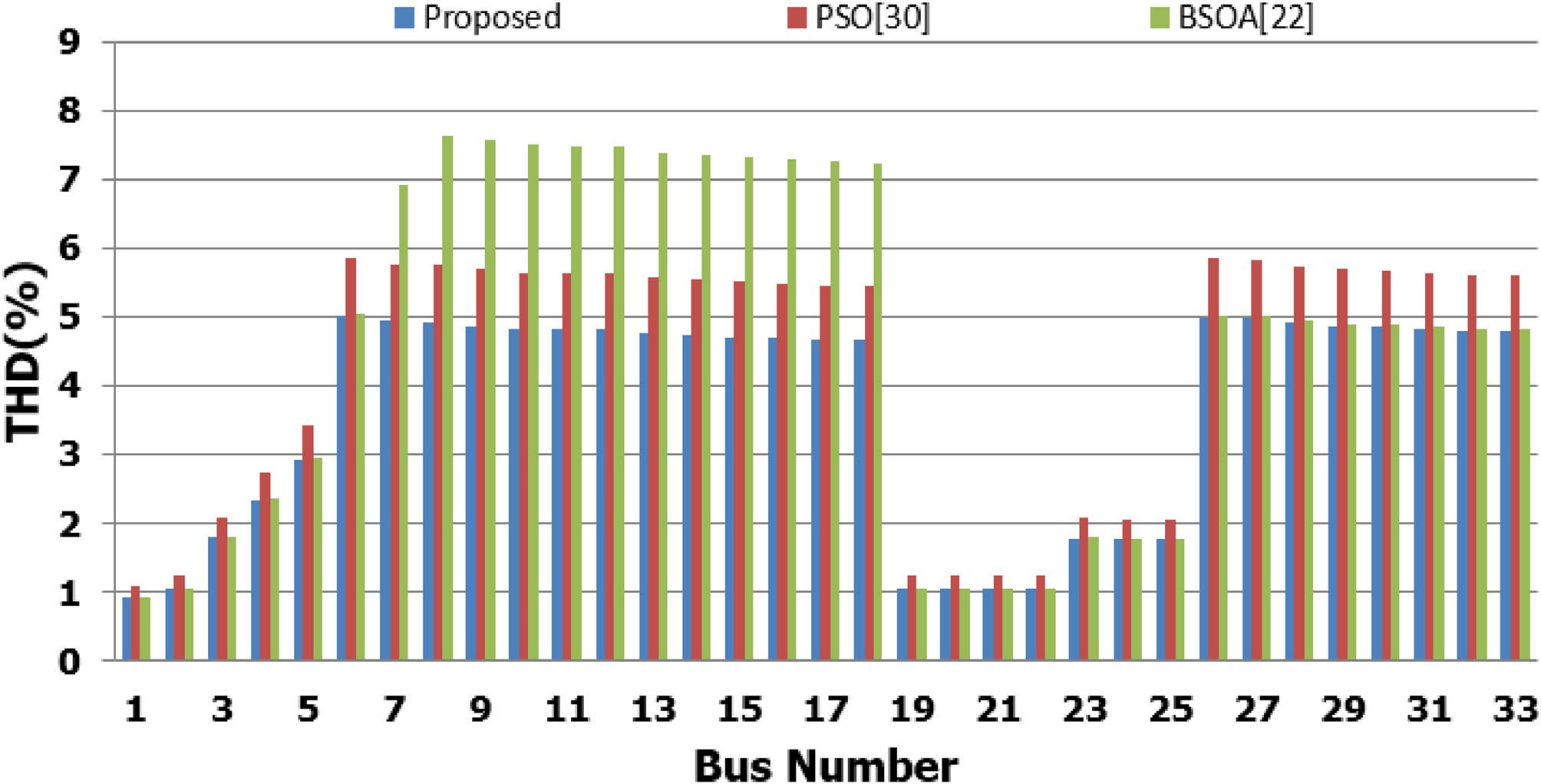
THD comparison for IEEE-33 bus system during base load operation.

In order to assist the effectiveness of the proposed methodology, the system performance using the proposed methodology of the first stage is compared to the reported works in^22,30^, as shown in Table 9. The authors of ref^30^ have recommended a DG of size 2588.4 kW with a unity power factor at bus number 6, while the authors of^22^ have suggested a DG of size 1857.5 kW at 0.82 power factor (DG has active and reactive power capability) connected to bus number 8. Table 9 shows that all the presented works reduce the system losses and voltage deviation compared to operation without DG. However, the total real power losses are 109.89 kW, 79.44 kW, and 76.282 kW using PSO^30^, BSOA^22^ and the proposed method, respectively. Hence, the proposed method achieves fewer power losses. With the DG harmonic spectrum given in Table 3, the BOSA harmonics violate the permissible standard limits for maximum individual (3.3031%), and maximum total harmonic distortion (7.6171%) and the PSO results violate the permissible standard with respect to maximum total harmonic distortion (5.8189%). On the other hand, the proposed strategy maintains both the maximum individual and total harmonic distortion within the permissible standard, with values of 2.17% and 5%, respectively. Eventually, the proposed strategy achieves minimum active power losses, minimum reactive power losses, and maintains both the voltage and harmonics within acceptable limits as shown in Fig. 24.

**Table 9:** Performance comparison for IEEE 33-bus system during base linear load operation with different algorithms.

	Without DG	PSO^30^	BSOA^22^	Proposed
DG size (kW)	–	2588.4	1857.5	1799.1
DG power factor	–	1	0.82	0.813
DG Bus NO	–	6	8	6
Fundamental ${P}_{losses}(kW)$	203	104	73	72
Fundamental ${Q}_{losses}(kVAR)$	140	70	50	50
Harmonic ${P}_{losses}(kW)$	0	5.89	6.44	4.282
Harmonic ${Q}_{losses}(kVAR)$	0	33.20	38.08	24.401
Total ${P}_{losses}(kW)$	203	109.89	79.44	**76.282**
Total ${Q}_{losses}(kVAR)$	140	103.2	88.08	**74.401**
${V}_{min}(P.U)$	0.913@ bus18	0.951 @ bus18	0.953 @ bus18	0.952 @ bus18
${V}_{max}(P.U)$	1@ bus1	1 @ bus1	1 @ bus1	1 @ bus1
${\Delta{V}}_{max}\left(\%\right)$	8.77	4.9	4.7	4.8
Max IHD (%)	0	2.5275	3.3031	**2.17**
Max THD (%)	0	5.8189	7.6171	**5**

Significant values in bold.

## System performance according to IEEE 1547–2018

This section briefly discusses the optimal allocation of DG considering the permissible harmonic distortion according to the IEEE 1547–2018 standard as shown in Table 10^41^. It is clear that the amount of harmonic generation from the distributed generator, according to IEEE 1547-2018, becomes less than ref^39^. This reduction in harmonics increases the generation system’s hosting capacity to accommodate extra generation, resulting in less power losses and less voltage deviation. For the base case with linear load, the obtained optimal DG is 2.544 MW with 0.824 power factor @ bus 6 as shown in Table 11.The new standard reduces the system losses from 76.82 to 61.57 kW, in addition the maximum voltage deviation is enhanced from 4.8 to 3.3 V. With total harmonics enhancement from 5% to 2.1499% and individual harmonics improvement from 2.17 to 0.633%. Therefore, the new standard provides several benefits.

**Table 10:** Inverter-based DG harmonics according to IEEE 1547 standard41.

Harmonic Order	${I}_{spectrum}^{h}\left(\%\right)$	Harmonic Order	${I}_{spectrum}^{h}\left(\%\right)$
1	100	29	0.6
5	4	31	0.6
7	4	35	0.3
11	2	37	0.3
13	2	41	0.3
17	1.5	43	0.3
19	1.5	47	0.3
23	0.6	49	0.3
25	0.6

**Table 11:** Comparison of optimal DG performance at base case with harmonic spectrum according to^39^ and IEEE 1547 standard^41^.

	Without DG	With DG’s harmonics according to^39^	With DG harmonics according to IEEE 1547-2018
DG size (kW)	–	1799.1	2544.7
DG power factor	–	0.813	0.824
DG Bus NO	–	6	6
Fundamental ${P}_{losses}(kW)$	203	72	61
Fundamental ${Q}_{losses}(kVAR)$	140	50	50
Harmonic ${P}_{losses}(kW)$	0	4.282	0.57
Harmonic ${Q}_{losses}(kVAR)$	0	24.401	3.85
Total ${P}_{losses}(kW)$	203	76.282	**61.57**
Total ${Q}_{losses}(kVAR)$	140	74.401	**53.85**
${V}_{min}(P.U)$	0.913@ bus18	0.952 @ bus18	**0.967 @ bus18**
${V}_{max}(P.U)$	1@ bus1	1 @ bus1	1 @ bus1
${\Delta{V}}_{max}\left(\%\right)$	8.77	4.8	3.3
Max IHD (%)	0	2.17	0.6330
Max THD (%)	0	5	2.1499

Significant values in bold.

**Figure 25:**
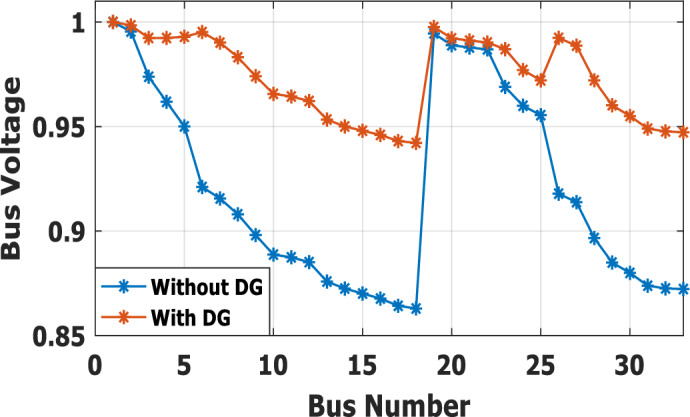
Voltage profile for heavy Load.

**Figure 26:**
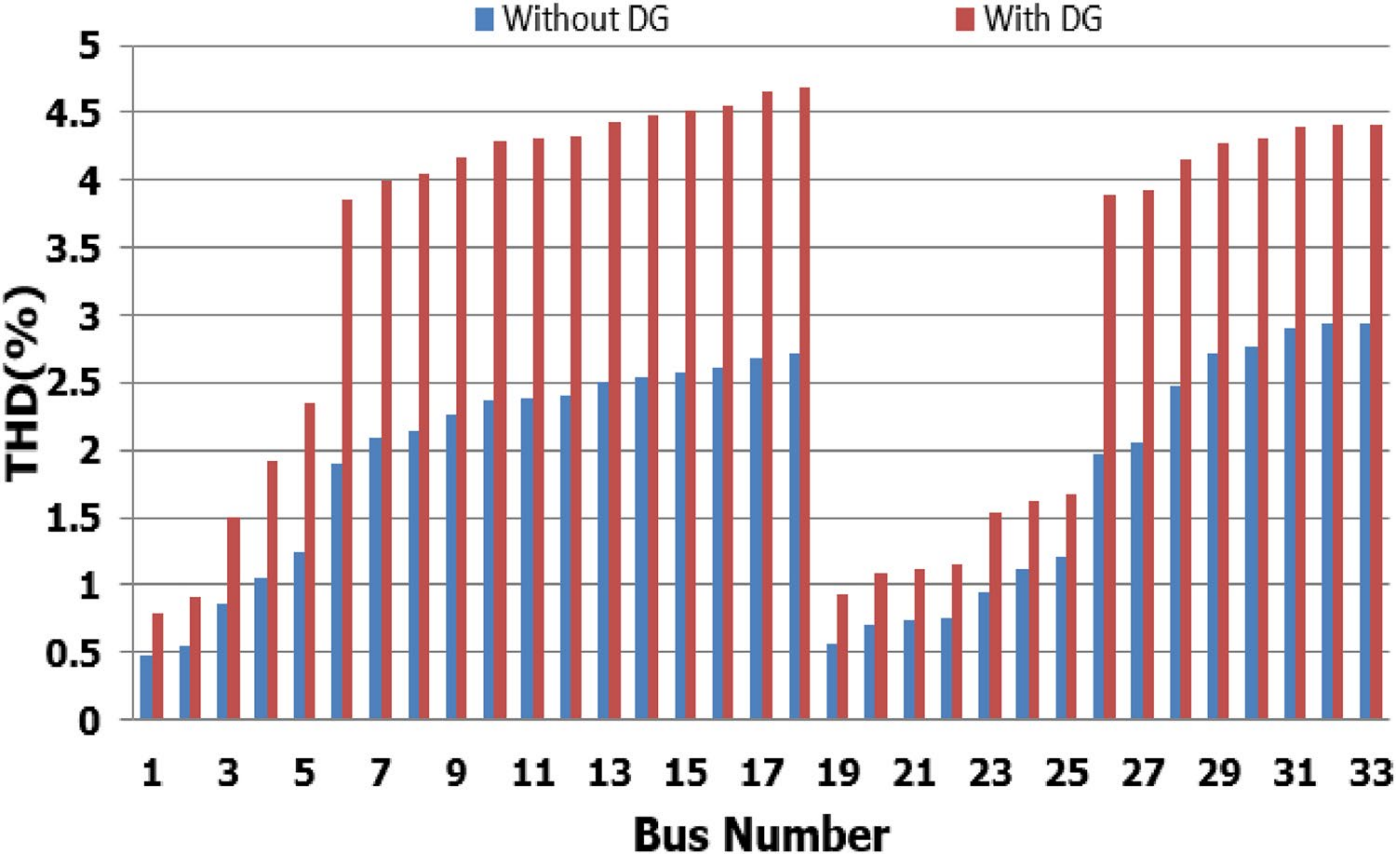
THD for heavy Load.

**Figure 27:**
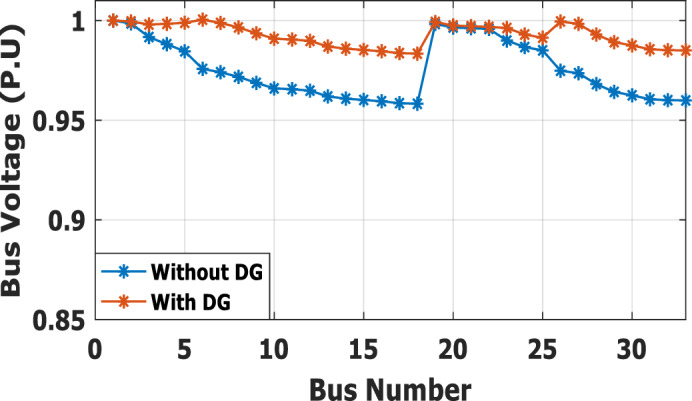
Voltage profile for light Load.

**Figure 28:**
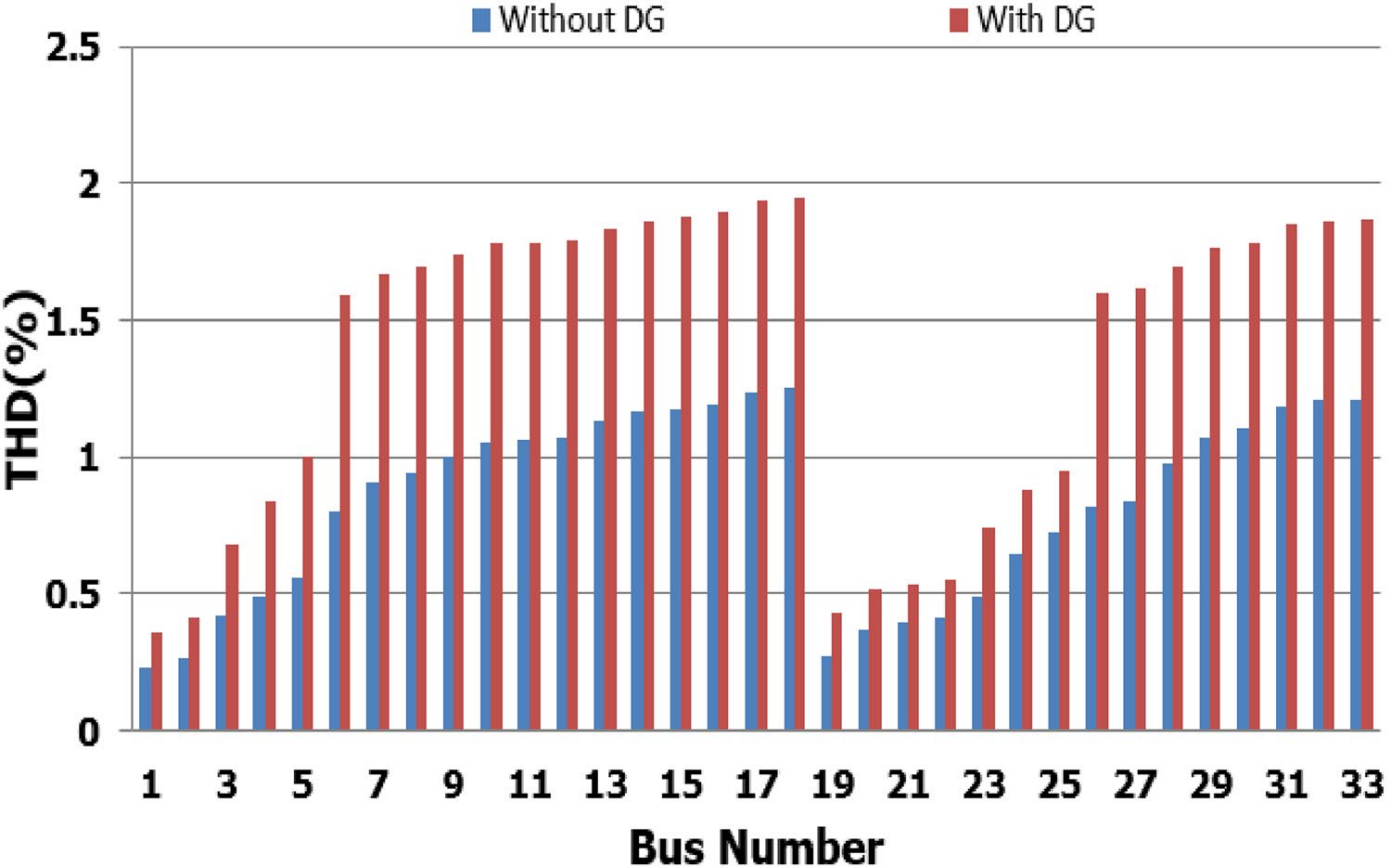
THD for light Load.

The second stage determines the optimal DG sizing while the DG is connected to bus 6 for ± 50% load change. At nonlinear load increase by 50% of the distribution network without DG, the minimum bus voltage is 0.863 p.u. at bus 18. The system has total active and reactive losses of 482.583 kW and 327.765 kvar, respectively. With DG at bus 6 with a rating of 3.412 MW and 2.559 Mvar, the minimum voltage becomes equal to 0.942 p.u. So, the voltage profile is improved, as shown in Fig. 25. Also, the real and reactive power losses are reduced to 148.903 kW and 130.087 kvar, respectively. The maximum IHD is 2.828% and the maximum THD is 4.6903%, as presented in Fig. 26, which is inside the standard limits. In case the total load decrease by 50% with the same DG location, the optimal rating for DG becomes 1.24 MW and 0.866 Mvar. The minimum bus voltage at bus 18 has improved from 0.958 to 0.983 p.u., as shown in Fig. 27. Also, the real and reactive power losses are reduced from 47.294 kW to 15.692 kW and 31.554 to 13.748 kvar, respectively, compared to without DG penetration. On the other hand, the proposed strategy is able to maintain both IHD and THD within the permissible standard with values of 1.1453% and 1.9446%, respectively, as presented in Fig. 28. Table 12 summarizes the system performance during both heavy load (50% load increase) and light load (50% loading decrease) scenarios using DG of stage 2. The recommended DG size to optimize the system and keep the system variable within safe boundaries during different loading scenarios is (3.412 MW, 2.559 Mvar) at bus 6.

**Table 12:** Performance comparison for IEEE-69 bus system during different cases of load.

DG rating (kVA)	Load increase by 50%		Load decrease by 50%	
	Without DG	3412.268 + 2559.201i (stage 2)	Without DG	1240.488 + 865.866i (stage2)
Bus NO	–	6	–	6
Fundamental ${P}_{losses}(kW)$	481	145	47	15
Fundamental ${Q}_{losses}(kVAR)$	320	110	30	10
Harmonic ${P}_{losses}(kW)$	1.583	3.903	0.294	0.692
Harmonic ${Q}_{losses}(kVAR)$	7.765	20.087	1.554	3.748
Total ${P}_{losses}(kW)$	482.583	148.903	47.294	15.692
Total ${Q}_{losses}(kVAR)$	327.765	130.087	31.554	13.748
${V}_{min}(P.U)$	0.863	0.942	0.958	0.983
${V}_{max}(P.U)$	1	1	1	1
${\Delta{V}}_{max}\left(\%\right)$	13.7	5.8	4.2	1.7
Max IHD (%)	2.033	2.828	0.855	1.1453
Max THD (%)	2.941	4.6903	1.25	1.9446
The recommended DG size	**DG (3.412MW, 2.559 Mvar) @ bus 6**			

Significant values in bold.

## Computational time analysis

The optimization problem that deals with harmonic power flow is suffering from computational burdens due to solving the power flow problem over the frequency range under consideration in each iteration. In this article, the system simulations and optimization process are performed using MATLAB (R2016) platform integrated with MATPOWER tool and genetic algorithm toolbox, run on a Core i7 processor (3.3 GHz), 16-GB RAM laptop. The optimization process of the first and second stages for DG sizing and allocation takes around 120 min for a population size of 50 and a generations size of 200 (around 10,000 iterations). The first and second stages for DG sizing and allocation are carried out offline; hence, the time is not critical for these stages.

**Figure 29:**
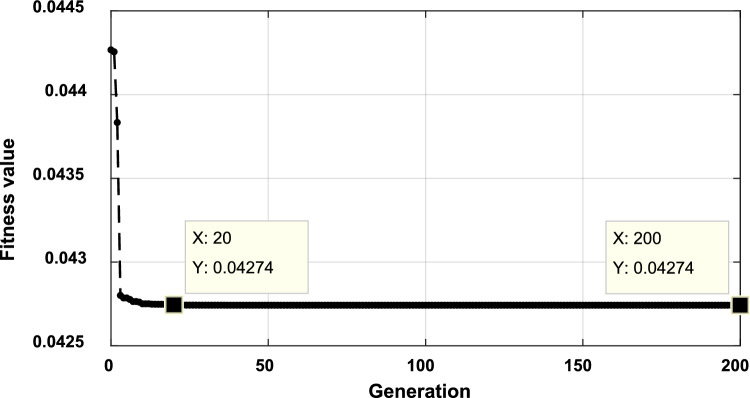
Convergence of the fitness function using population size of 50 and generation size of 200 (10,000 iterations) during 7:00 interval.

**Figure 30:**
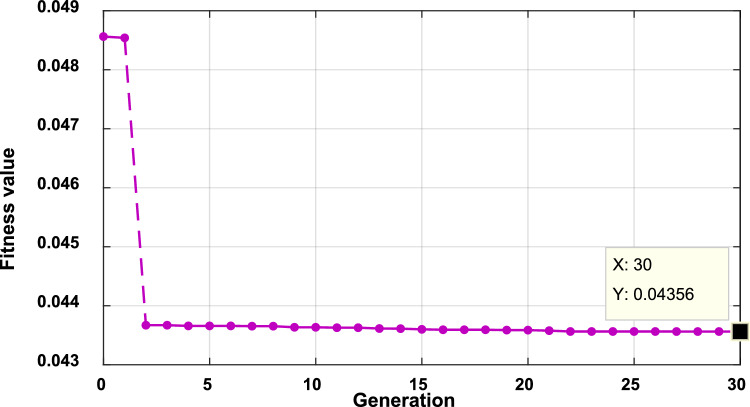
Convergence of the fitness function for population size of 20 and generation size of 30 (600 iterations) during 7:00 interval.

However, 120 min for optimal power dispatch optimization considering, the system harmonics is long for real-time or even day-ahead operation. Therefore, during the real-time operation of the third stage, the system computation time can be reduced using a higher computational device, reducing the population size, reducing the generation size, or ignoring the higher order harmonics. Also, a combination of these solutions can be used. For instant the convergence curve with population size of 50 and generation size of 200 (10,000 iterations) is shown in Fig. 29. It is clear, the optimization process reaches its optimal point after 20 generations (1000 iterations) with a total fundamental loss of 42.74 kW in around 120 min. While, when the population size is reduced to 20 and the generation size is reduced to 30 (600 iterations), the system achieves real power losses of 43.56 KW, as shown in Fig. 30, and takes around 20 min. It is clear, reducing the number of iterations achieves near optimal operation (43.56 KW losses instead of 42.74 KW), while the optimization time is reduced significantly (from 120 to 20 min), which is suitable for the day ahead, one hour ahead, or 30 min ahead.

## Conclusion and future research

A multi-stage framework is presented for optimizing the inverter-based distributed generator, such as fuel cell-based DG location and size, for enhancing the voltage profile and minimizing network power losses, considering harmonic pollution from both nonlinear loads and DG using a genetic algorithm. The proposed approach is applied to the IEEE 33-bus radial distribution system with a variety of linear and nonlinear load combinations.

Compared to operation without DG, DG integration in the IEEE 33-bus system and operation at base load reduce the real power losses from 203 to 109.89 kW, 79.44 kW, and 76.28 kW utilizing PSO^30^, BSOA22 and the proposed method, respectively. Hence, the proposed method achieves lower power losses with a slight reduction compared to BSOA. However, the BOSA harmonic content violates the allowable limits for both individual (3.3031%) and total harmonic distortion (7.6171%). Hence, optimizing inverter-based DG without addressing the harmonic pollution may enhance the voltage and reduce the power losses, while the harmonic pollution may exceed the permissible limits. The proposed strategy is able to maintain both individual and total harmonic distortion within the permissible standard, with values of 2.17% and 5%, respectively. Consequently, the proposed strategy achieves minimum active power losses and maintains both the voltage and harmonics within the allowable limits. Moreover, IEEE 1547-2018 provides restrictions on the DG’s manufacture to reduce inject current distortion, which increases the permissible DG penetration into the distribution network. As a result, the real power loss is reduced, voltage profile is enhanced.

The optimal DG location is bus 6 with a rating of 1.799 MW and 1.288 Mvar during linear loads (specified from the first stage), which enhances the maximum voltage deviation from 8.77 to 4.8%, reduces the power losses from 203 to 76.28 kW, and keeps the dividable and total harmonic distortion at acceptable levels. While at 50% nonlinear load increase, the individual and total harmonic distortions reach 3.99% and 6.45%, respectively, which exceed the standard boundaries. Hence, the DG shall be sized to cover the expected range of load variation. Therefore, for a wide range of nonlinear load penetration (50% load increase), the recorded active power of DG is raised to 2.097 MW, and the reactive power is decreased to 0.829 Mvar (specified from the second stage). It is clear that during high penetration of nonlinear loads, the required active power increases to provide more local generation, while the required reactive power decreases to avoid exceeding the harmonic distortion.

The optimal size and location of the DG are determined using the first and second stages, which ensure DG capability for optimizing system real losses without violating harmonic boundaries during a wide range of nonlinear load penetration. In a real system, the demand varies throughout the day. Therefore, the actual generation of the DG is controlled through the third stage of the proposed framework to ensure optimal operation without breaching the system constraints at any time of the day. For IEEE 33-bus, the third stage succeeded in controlling the DG active and reactive power generation throughout the day for loads varying from 0.5 pu to about 1.35 pu, while the voltage and harmonics were kept within the permissible limits. Which means the capability of the selected DG size, location, and proposed methodology to operate properly for future load growth. The proposed methodology is able to minimize the daily energy losses from 5.281 to 2.452 MWh/day, which means an energy saving of 53.6%.

Due to the importance and increasing integration of inverter-based distributed generators, this research can be extended to examine the proposed framework to elucidate multi-objective optimization, such as improving voltage stability, minimizing operation costs, reducing power losses, and enhancing the voltage profile while limiting harmonic distortion. In addition, the suggested methodology can be investigated for the optimal placement and sizing of multiple DG units in distribution systems that involve different types of nonlinear loads, such as electric vehicles, data centers,…etc. Furthermore, the suggested methodology can be studied for the optimal placement and sizing of multiple different renewable-based DG units in distribution systems such as photovoltaic and wind generation, considering their uncertainty and variability according to weather conditions. As well as consideration of energy storage systems. eventually, using the fast harmonic calculation technique to accelerate the optimization process with optimal results for real-time operation.

## Data Availability

All data generated or analyzed during this study are included in this published article.

## Abbreviations

DG: Distributed generators
GA: Genetic algorithm
VSI: Voltage stability index
PPF: Passive power filters
RMS: Root mean square
IHD: Individual harmonic distortion
THD: Total harmonic distortion
HPF: Harmonic power flow
PSO: Particle swarm optimization
BSOA: Backtracking search optimization algorithm
${P}_{d,i}$: Linear loads at fundamental frequency active power
${P}_{loss,k}$: Power losses of the individual branch
${P}_{loss,k}^{h=1}$: Fundamental power losses
${P}_{loss,k}^{h}$: Losses due to harmonics injection
$\left|{I}_{k}\right|$: Magnitude of current flows through branch $k$
${R}_{k}$: Resistance of branch $k$
${V}_{i}^{h=1}$: RMS values of the fundamental bus voltage
${V}_{i}^{h}$: RMS values of the bus voltage at harmonic order h
${V}^{min}$: Minimum acceptable voltage levels
${V}^{\text{max}}$: Maximum acceptable voltage levels
${V}_{rms\_i}$: RMS of bus voltage
${P}_{g}$: Utility grid real power
${P}_{Di}$: Demand load at bus $i$
${P}_{DG}$: DG injected real power
${P}_{Losses}$: Active power losses
${Q}_{g}$: Total injected reactive power into the distribution system from the utility
${Q}_{ DG}$: Total injected reactive power into the distribution system from the DG
${Q}_{Di}$: Total reactive power of the demand
${Q}_{loss,k}$: Reactive power losses of branch $k$
${Q}_{loss,k}^{h=1}$: Fundamental reactive power losses
${Q}_{loss,k}^{h}$: Total harmonic reactive power losses
${\left|{I}_{k}\right|}_{max}$: Maximum allowable current of the branch $k$
$\left|{I}_{k}\right|$: RMS Current of the branch $k:$
${IHD}_{\text{i}}(h)$: Individual Harmonic distortion
${IHD}_{max}\left(h\right)$: Maximum allowable individual harmonic distortion of order $h$
${THD}_{max}$: Maximum permissible total harmonic distortion
${P}_{gen i,min}$: Minimum permissible generation active power at bus $i$
${P}_{gen i,max}$: Maximum permissible generation active power at bus $i$
${Q}_{gen i,min}$: Minimum generation reactive power at bus $i$
${Q}_{gen i,max}$: Maximum generation reactive power at bus $i$
$$h$$: Harmonic order
${z}_{ij}^{h}$: Branch impedance between bus $i and j$
${x}_{ij}$: Branch’s reactance at the fundamental frequency
$${r}_{ij}$$: Branch resistance
${y}_{ij}^{h}$: Branch admittance between bus $i and j$
${Q}_{d,i}$: Linear loads at fundamental frequency reactive power
${r}_{d,i}and{x}_{d,i}$: Equivalent resistance and fundamental equivalent reactance
${y}_{load,i}^{h}$: Equivalent admittance of linear load at bus $i$ for harmonic order $h$.
${I}_{nlj,i}^{h=1}$: Fundamental injected current of the nonlinear load.
${P}_{nl,i}and{Q}_{nl,i}$: Nominal real and reactive power of the nonlinear load at bus $i$
$\left|{I}_{nlj,i}^{h=1}\right|$: RMS of the injected current at fundamental frequency
$\left|{I}_{nlj,i}^{h}\right|$: RMS of the injected current at harmonic order $h$
$${I}_{spectrum}^{h}$$: Current spectrum
${\theta }_{spectrum}^{h}$: Phase shift spectrum
${\theta }_{nlj,i}^{h=1}$: Phase shift of the injected current at the fundamental frequency
${\theta }_{nlj,i}^{h}$: Phase shift of the injected current at the harmonic order $h$
${I}_{DG,i}^{h=1}$: Fundamental injected current of the DG
${I}_{D{G}_{spectrum}}^{h}$: DG harmonic current magnitude spectrum
${\theta }_{DG\_spectrum}^{h}$: DG harmonic current phase shift spectrum
${\theta }_{DG,i}^{h=1}$: Fundamental current phase shift.
${Z}_{grid}$: Grid internal impedance
${S}_{sc}$: Grid short circuit level
${V}_{n}$: Nominal network voltage
$${X}_{grid}$$: Grid reactance
$${R}_{grid}$$: Grid resistance
${\text{V}}_{\text{bus}}^{\text{h}}$: Vector of bus voltages
${\text{I}}_{\text{bus}}^{\text{h}}$: Vector of bus injection currents
$\left|{I}_{nlj,i}^{h=1}\right|$: RMS of the injected current at fundamental frequency
${\text{Z}}_{\text{bus}}^{\text{h}}$: Impedance matrix of the distribution system
${\Delta{V}}_{max}$: Maximum voltage deviation in the network
${THD}_{\text{i}}$: Total harmonic distortion at bus $i$
